# Poor prey quality is compensated by higher provisioning effort in passerine birds

**DOI:** 10.1038/s41598-021-90658-w

**Published:** 2021-05-27

**Authors:** Sarah Senécal, Julie-Camille Riva, Ryan S. O’Connor, Fanny Hallot, Christian Nozais, François Vézina

**Affiliations:** 1 Département de biologie, chimie et géographie, Groupe de recherche sur les environnements nordiques BORÉAS, Université du Québec à Rimouski, Rimouski, QC Canada; 2 Center for Northern Studies, Université du Québec à Rimouski, Rimouski, QC Canada; 3 Quebec Center for Biodiversity Science, McGill University, Montreal, Canada; 4 Québec Océan, Université Laval, Quebec, Canada

**Keywords:** Behavioural ecology, Ecophysiology

## Abstract

In altricial avian species, nutrition can significantly impact nestling fitness by increasing their survival and recruitment chances after fledging. Therefore, the effort invested by parents towards provisioning nestlings is crucial and represents a critical link between habitat resources and reproductive success. Recent studies suggest that the provisioning rate has little or no effect on the nestling growth rate. However, these studies do not consider prey quality, which may force breeding pairs to adjust provisioning rates to account for variation in prey nutritional value. In this 8-year study using black-capped (*Poecile atricapillus*) and boreal (*Poecile hudsonicus*) chickadees, we hypothesized that provisioning rates would negatively correlate with prey quality (i.e., energy content) across years if parents adjust their effort to maintain nestling growth rates. The mean daily growth rate was consistent across years in both species. However, prey energy content differed among years, and our results showed that parents brought more food to the nest and fed at a higher rate in years of low prey quality. This compensatory effect likely explains the lack of relationship between provisioning rate and growth rate reported in this and other studies. Therefore, our data support the hypothesis that parents increase provisioning efforts to compensate for poor prey quality and maintain offspring growth rates.

## Introduction

For altricial bird species that actively provision nestlings, access to high-quality food can significantly impact offspring’s future survival by allowing nestlings to fledge early and gain access to high-quality territories^1–5^. Nestlings with greater mass at fledging also have higher chances of survival and increased future reproductive opportunities^6–8^. Thus, the strategy used to exploit food resources and the effort invested by parents in supplying offspring with high-quality food likely affect the future survival of offspring^1–3,9^. Consequently, nestlings’ fitness should be linked to interrelated effects of resource availability, parental foraging effort and provisioning rate, but there is very little data supporting this.

Nest visitation rate is commonly interpreted as a measure of provisioning effort, and it is often assumed that a higher provisioning (or visitation) rate by parents should be positively related to breeding productivity and reproductive success (see^10^ for a review). However, recent studies have shown large individual variation in parental provisioning rates and a general absence of a correlation between reproductive success indicators (e.g. number of nestlings fledged, fledging mass) and the number of feeding visits made by parents^8,10–19^. For instance, in European starlings (*Sturnus vulgaris*), no relationship was found between provisioning rate and productivity (assessed by brood size at fledging and mean fledgling mass), suggesting that brood development rate and overall reproductive success were affected by factors other than provisioning rate alone^10,20^. Ultimately, daily energy and nutrient input provided to nestlings should be critical elements affecting growth rate and, importantly, both factors may vary independently from parents’ provisioning rate. Individual variation in provisioning rate could thus reflect different foraging strategies by adults attempting to exploit prey of varying nutritional quality. However, most studies have not considered relationships between components of parental effort, such as provisioning rate and the quality of prey provisioned to nestlings (but see^1,2^).

This study aimed to determine the relationship between breeding productivity and quality of prey (i.e., energy value) provided to nestlings across years and whether adults adjust their provisioning rate to accommodate for yearly variation in prey quality, thus explaining the lack of a relationship between nestling growth rate and provisioning rate often found in previous studies (e.g.^10,20^). We used black-capped (*Poecile atricapillus*) and boreal (*Poecile hudsonicus*) chickadees as model species. Both species reproduce in artificial nest boxes and have similar reproductive characteristics such as growth rate, number and mass of nestlings (see Supplementary material).

We first documented the relationship between parental provisioning rate and nestling growth rate across years. We then investigated the relationship between prey quality and nestling growth rate and assessed whether variation in provisioning rate correlated with prey quality. As we hypothesized that adults would adjust their provisioning rate to compensate for prey of different energetic value, we predicted that nestling growth rates would not correlate with provisioning rates but would increase with prey energy value. Specifically, we expected higher provisioning rates and that greater amounts of food would be brought to nestlings when prey energetic value was low, and conversely, lower provisioning rates would be observed and lower amounts of food would be brought when prey of greater energetic value were consumed. Since adults were expected to adjust provisioning rates to prey quality, we also predicted the nestling growth rate to be maintained across years.

## Methods

### Breeding data

This study was carried out near Rimouski, Québec, Canada, in the Forêt d’enseignement et de recherche Macpès (48°18′24.8"N, 68°31′44.7"W). From 2011 to 2019, we closely monitored breeding black-capped and boreal chickadees using artificial nest boxes. This included data for 858 nestlings from 173 broods (98 black-capped chickadees, 75 boreal chickadees). The first eggs are typically laid in May (mean lay date: May 25, range: May 8 to June 14), with nestlings from the first brood leaving the nest in July (mean fledge date: July 2, range: June 17 to July 20). From the day of hatching (herein, day 1 of the nestling period) up to day 15, nest boxes were visited every 24 h (± 37 min) to weigh (± 0.01 g) each nestling and determine daily growth rates (g day^−1^). We calculated the daily growth rates by subtracting the mass of the preceding day (M_*x*-1_) from the mass of the current day (M_*x*_; thus, the daily growth rate in g day^−1^ = M_*x*_ − M_*x*-1_ adjusted to the exact time-interval between measures to have a growth rate per 24 h). We then averaged the growth rate of all nestlings from the same brood to obtain a mean growth rate per brood (mean g day^−1^ for all nestlings of a brood). Nestlings from the same nest were differentiated from each other by clipping the tip of the claw from a specific digit. On day 13, nestlings were fitted with a metal ring and a unique combination of plastic-colored rings. Beginning on day 17, nest boxes were checked daily to determine the fledging date, which usually occurred around day 18 (mean ± SD fledge day: 18.61 ± 0.99). Methods were approved by the animal care committee of the Université du Québec à Rimouski (CPA-69–17-90) and have been conducted under scientific and banding permits from Environment Canada–Canadian Wildlife Service and are reported in accordance with ARRIVE guidelines. All methods were carried out in accordance with relevant guidelines and regulations.

### Provisioning rate

From 2014 to 2019, both male and female breeders were captured in nest boxes on the afternoon of day 13 by blocking the entrance of the nest box with a sponge attached to a fishing line going through the box (see^21^ for detailed procedure). Parents were not captured before this day as they are known to abandon the nest if captured too early (Vézina, pers. comm.). Upon capture, adults were sexed by the presence of either a cloacal protuberance (male) or a brood patch (female) and were ringed with a US Geological Survey numbered metal ring, one colored plastic ring, and a passive integrated transponder (PIT) tag embedded within another colored plastic ring (2.3 mm EM4102, Eccel Technology Ltd, Groby, Leicester, UK). A total of 134 adults were tagged. Each nest box was fitted with a radio-frequency identification system (RFIDLog, Priority 1 Design, Melbourne, Australia), including an SD card and an antenna positioned at the nest box entrance to measure provisioning rates by each parent until fledging. The RFID system registered a PIT-tag every time a bird passed through the antennae^21,22^. Since the raw data included both the entering and exiting of nest boxes, we divided the total number of detections by two to have only the number of provisioning visits as our measure of provisioning rate (i.e., effort). Parents were assumed to provision whenever they visited their nest (and field observations confirmed food in the beak in all cases). As parents were banded with a PIT-Tag on day 13 after hatching, we then computed the mean number of visits per day from day 14 until the last day before fledging to use in provisioning rate analyses. Although the provisioning rate increases with nestling age, it is also highly repeatable and positively correlated across ages within broods^20^ (see Supplementary material for an analysis on a smaller sample comparing provisioning data before day 13 to day 14–20 within brood, R^2^ = 0.85, P < 0.001). Our measure of daily mean provisioning rates over days 14–20 is, therefore, representative of the provisioning rate of parents over the entire nestling period (see Supplementary material). Provisioning rate values were averaged per breeding pair for analyses.

### Prey quality

We used the energy content of ingested prey (arthropods including larvae and spiders) by nestlings as a proxy for prey quality. Prey was obtained using a commonly implemented stomach flushing technique which provides ingested food samples from live birds without harming the animal^23–28^. This technique allows for repeated sampling and is efficient to empty a birds’ stomach^23^. Stomach contents were collected from each nestling between 2017 and 2019. Stomach flushing were performed in the morning (between 8:30 am and 11:30 am) on days 8 (which corresponds to peak daily growth rate in our population, see results), 10 and 12, at which time nestling digestive systems should match that of adults in size^29^. Nestlings were removed from nest boxes immediately after a provisioning visit by an adult and weighed. Each nestling was then held in the hand, and a flexible surgical plastic tube (size 18.5 gauge for < 7 g nestlings and 14.4 gauge for ≥ 7 g nestlings) previously disinfected with chlorhexidine, rinsed in water and coated with Vaseline, was passed down the esophagus and into the gizzard, at which point warm water was injected (1.0 mL) slowly into the tube with a syringe. At the first sign of regurgitation, the injection of water stopped, and the tube was gently removed. The process was conducted over a funnel to collect all stomach contents into a Nalgene container. Nestlings were then returned to their nest box. The procedure lasted on average 18 ± 12 min per brood. All samples were kept on ice before being stored in a − 80 °C freezer until laboratory analyses. A total of 1036 samples were collected from 84 broods (168 black-capped chickadee nestlings, 114 boreal chickadee nestlings).

We used bomb calorimetry to determine the energetic value of stomach contents. Each sample (three per nestling, representing days 8, 10, and 12) was filtered individually using a 20 µm filter and left to air dry overnight. The filters were weighed before and after use to obtain the dry mass of samples. Since our samples had very low mass (mean ± SD average sample mass = 2.90 ± 3.70 mg), we had to pool samples from a brood for each day of stomach flushing to obtain precise measurements (samples have to be over 2 mg for the equipment to work properly). This resulted in three samples per brood (days 8, 10, and 12). Those samples (10.07 ± 10.68 mg) were then each combined with a known amount of benzoic acid (a standard of known calorific value) to create pellets, which were then burned in a micro bomb calorimeter (1109A vessel with the 6725 semi-micro oxygen bomb from Parr Instrument Company, Moline, Illinois, USA) to obtain their energy content in joules mg^−1^. We then subtracted the amount of energy released by the benzoic acid standard to obtain that of the original samples.

### Statistical analyses

In this study, we pooled the species data to avoid duplication of results because nestling mass and growth rate patterns, as well as parental provisioning rate and stomach content (quality and dry mass), did not differ significantly between the two species (see Supplementary material for more information). This was first confirmed by running models including species as a fixed parameter. However, as this variable was never significant, we removed it from models and presented here results using pooled data.

All analyses were performed in R Studio (3.6.1). We ran linear mixed-effects models, using the package “lme4”^30^, or multiple regression using the package “lmtest”^31^. Results (ANOVA tables) were obtained using “lmerTest”^32^ for each model separately and are presented for full models. For pairwise comparisons of means, we used the “emmeans” package^33^ to compute Tukey’s honestly significant difference (HSD). All presented data are shown as means ± standard deviation unless otherwise stated.

#### Interannual variation in growth rate, provisioning rate and prey quality

We began our analyses by comparing nestlings’ growth rate (g day^−1^ averaged for all nestlings of a pair for each day between days 2 and 15 of the nestling period), parental provisioning rate (visits day^−1^ pair^−1^ averaged for days 14–20), prey quality (J mg^−1^ per brood), and dry mass of stomach samples (quantity of prey matter per brood, mg) among years as follow.

Nestling growth rates were measured over eight breeding seasons (2011–2019, excluding 2016, where a separate investigation including brood manipulation took place, see^21^). We modelled yearly variation in nestling’s growth rate by fitting a linear mixed-effects model with year, age of nestlings (from 1 to 14) and brood size (from 1 to 8) as categorical fixed effects, and pair identity (a unique identifier for each brood) as a random factor. We used data from 858 nestlings from 173 broods (98 of black-capped chickadees, 75 of boreal chickadees) for this analysis. We then used pairwise comparisons of means (Tukey’s HSD) to test whether nestling growth rates differed among years.

Provisioning rates were calculated from 2014 (first use of RFID system) to 2019 (excluding 2016), and we used the mean number of visits per day over days 14–20 as an indicator of parental provisioning effort at the pair level (see Supplementary material). To investigate yearly variation in provisioning rate, we constructed a multiple regression model including mean provisioning rate as a dependent variable and year (2014–2019 excluding 2016) and brood size (1–8 nestlings) as categorical fixed factors. As the mean provisioning rate was used for this analysis, each breeding pair was present only once in the data set. Therefore, the inclusion of pair identity as a random factor was not required for this analysis. We used data from 73 broods (39 black-capped chickadees, 34 boreal chickadees). We used pairwise comparisons of means (Tukey’s HSD) to test whether provisioning rates differed among years.

We assessed yearly variation in prey quality as well as the dry mass of stomach contents from 2017 to 2019 by fitting two linear mixed-effect models (one for each variable) with year, age of nestlings for each stomach flushing (8, 10, or 12) and brood size (1–8 nestlings) as categorical fixed factors. We also included Julian date as a continuous covariate in models to control for the known influence of intra-seasonal variation in temperature on insect growth rate and abundance, potentially leading to heavier and more numerous prey items being provisioned by adults on warmer days^7,34–37^. Those models further included pair identity (a unique identifier for each brood) as a random factor. We used data from 63 broods (39 broods black-capped chickadees, 24 boreal chickadees) for these analyses. We used pairwise comparisons of means (Tukey’s HSD) to test whether prey quality and dry mass of stomach samples differed among years.

#### Inter-relation between growth rate, provisioning rate and prey quality across years

In a second step, we examined relationships between nestling growth rate, parental provisioning rate, prey quality, and quantity across years.

To assess whether the growth rate per brood varied with provisioning rate, we fitted a linear mixed-effect model with provisioning rate as a continuous variable and brood size (1–8 nestlings) as a categorical fixed factor. We also included the year (2014–2019, excluding 2016) as a random factor (one measure per breeding pair). We used data from 73 broods (39 black-capped chickadees, 34 boreal chickadees) for this analysis.

We examined the influence of prey quality (averaged for all three sampling days) on nestling growth rates by testing the effect of the stomach samples’ energy content on the brood’s growth rate. The linear mixed-effect model also included brood size (1–8 nestlings) as a categorical fixed factor and year (2017–2019) as a random factor (one measure per breeding pair). These analyses were based on data from 58 broods (36 black-capped chickadees, 22 boreal chickadees).

The relationship between provisioning rate and prey quality was investigated using a linear mixed-effect model to test the effect of stomach samples’ energy content on provisioning rate. The model also included brood size (1–8 nestlings) as a categorical fixed factor and year (2017–2019) as a random factor (one measure per breeding pair). For this, we used data from 41 broods (24 black-capped chickadees, 17 boreal chickadees).

We then determined whether the amount of food in nestlings’ stomachs (averaged across the three samples) was related to prey quality. In this case, the linear mixed-effect model tested for an effect of prey quality on the dry mass of stomach contents. The model included brood size (1–8 nestlings) as a categorical fixed factor and year (2017–2019) as a random factor (one measure per breeding pair). This analysis was based on data from 58 broods (36 black-capped chickadees, 22 boreal chickadees).

## Results

### Interannual variation in growth rate, provisioning rate and prey quality

**Figure 1:**
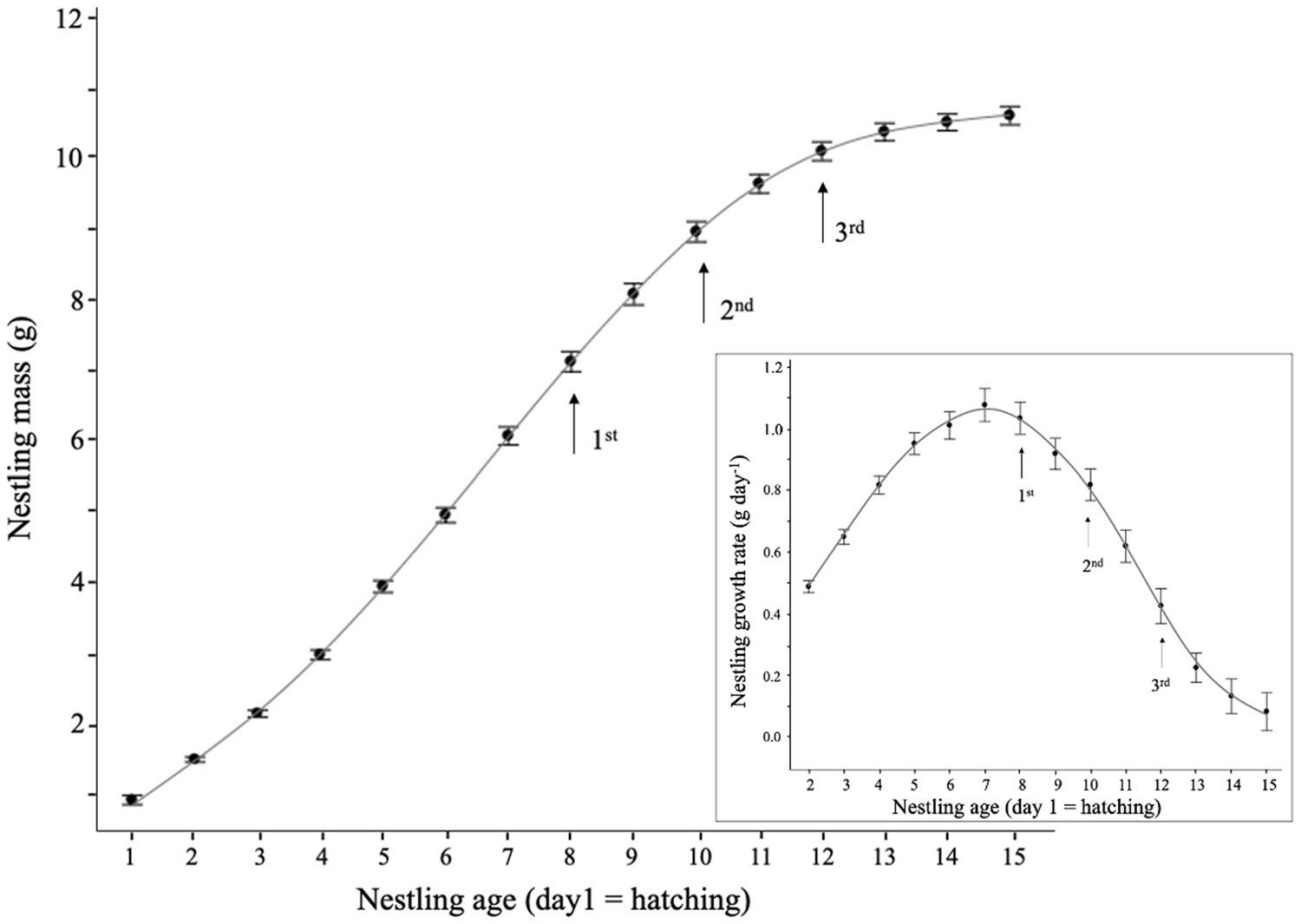
Growth curve showing nestling mean daily mass according to age in days since hatching in black-capped chickadees and boreal chickadees sampled at the FER Macpès, Rimouski, Canada. This figure shows both species combined (see text). The three arrows show the days of stomach contents sampling. Insert shows daily growth rate (calculated over the last 24 h) according to nestling age. We used data from 858 nestlings from 173 broods. Of those, 98 broods were black-capped chickadees and 75 broods were boreal chickadees. Data are mean ± 95% confidence interval.

**Figure 2:**
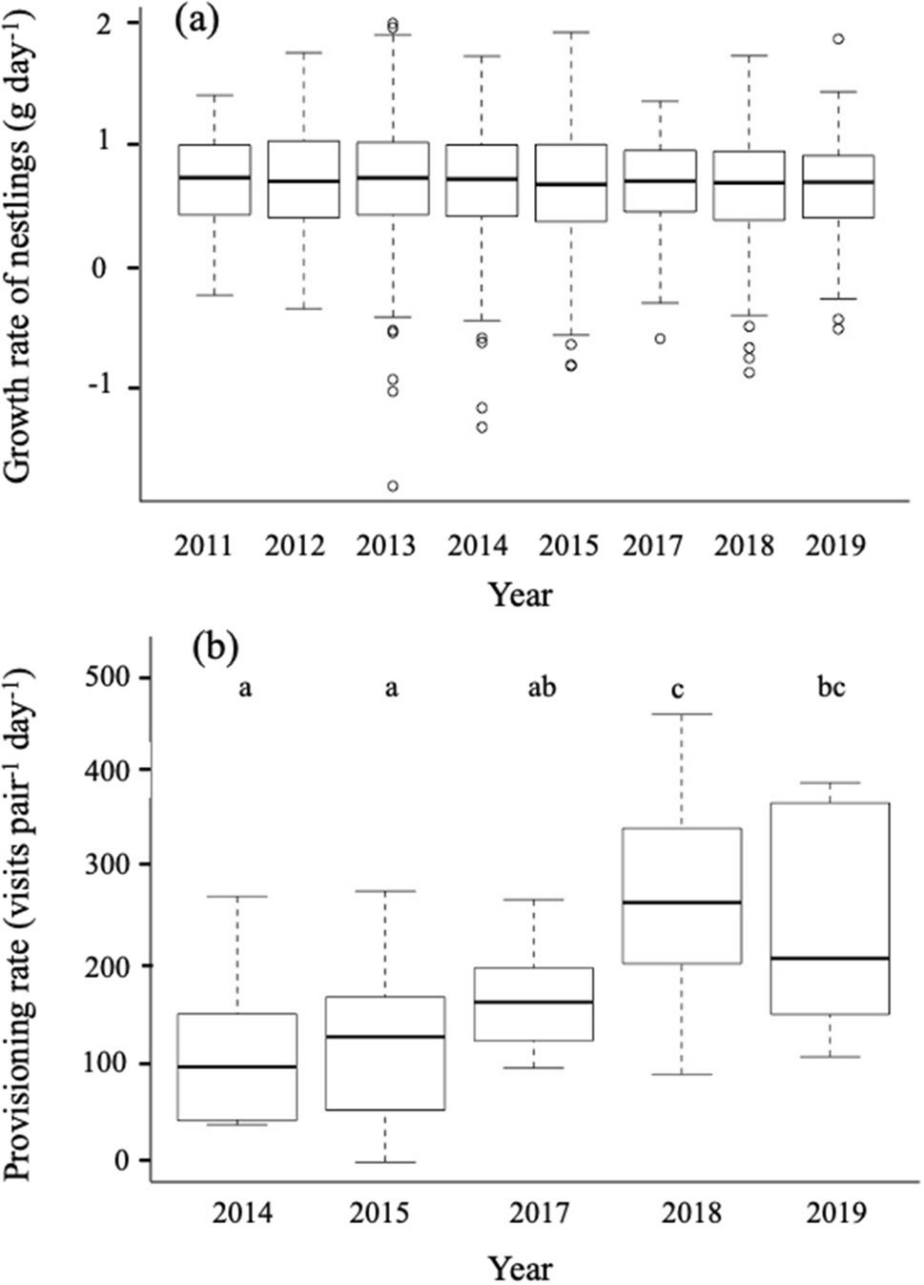
Yearly variation in nestling’s growth rate (**a**) and provisioning rate (**b**) in black-capped chickadees and boreal chickadees sampled at the FER Macpès, Rimouski, Canada (data combined for both species, see text). The dark line in boxes represents the median, the boxes show the interquartile range, the error bars represent minimum and maximum values and the points are extreme values. Data for yearly variation in nestling’s growth rate (**a**) included 858 nestlings from 173 broods. Of those, 98 broods were black-capped chickadees and 75 broods were boreal chickadees. Data for yearly variation in provisioning rate (**b**) included 73 broods. Of those, 39 broods were black-capped chickadees and 34 broods were boreal chickadees. Growth rate did not differ among years while provisioning rate was at its highest in 2018. Different letters in (**b**) indicate significant differences among years.

Nestling growth rate did not differ significantly among years (F_7, 104_ = 1.03, P = 0.4; Fig. 2a), with an average growth rate of 0.66 ± 0.43 g day^−1^ over all years. Brood size influenced growth rate (F_7, 218_ = 5.38, P < 0.001), but only for broods of either 1 or 8 nestlings, where the growth rate was lower (5 cases out of 173 broods; same daily growth rate of 0.67 ± 0.52 g day^−1^ on average for broods of 2–7 nestlings compared to 0.44 ± 0.42 g day^−1^ for broods of 1 and 8 nestlings) (Tukey: F_1,325_ = 8.295, P = 0.08). Daily growth rate did differ with age however (F_13,1977_ = 210.39, P < 0.001), increasing from day 2 to its peak (1.05 ± 0.35 g day^−1^) on day 7 (Fig. 1, Tukey: F_1,1965_ = 356.15, P < 0.001), although the rate on day 7 did not differ significantly from rates recorded on days 6 (Tukey: F_1,1951_ = 4.64, P = 0.6) and 8, (Tukey: F_1,1948_ = 2.03, P = 0.9). Daily growth rate then decreased from day 7 until the end of our measurement period on day 15. The lowest recorded rate of growth occurred during day 15, with a mean growth rate of 0.09 ± 0.37 g day^−1^ (Tukey: F_1,2096_ = 818.99, P < 0.001).

Mean provisioning rate varied significantly among years (F_4, 61_ = 9.49, P < 0.001), with a minimum in 2014 (119.08 ± 86.73 visits day^−1^) and a maximum, 2.3 times higher, in 2018 (273.97 ± 88.19 visits day^−1^, Tukey: F_1,61_ = 13,09, P < 0.005, Fig. 2b). Provisioning rate did not vary significantly with brood size (F_7, 61_ = 1.87, P = 0.09).

**Figure 3:**
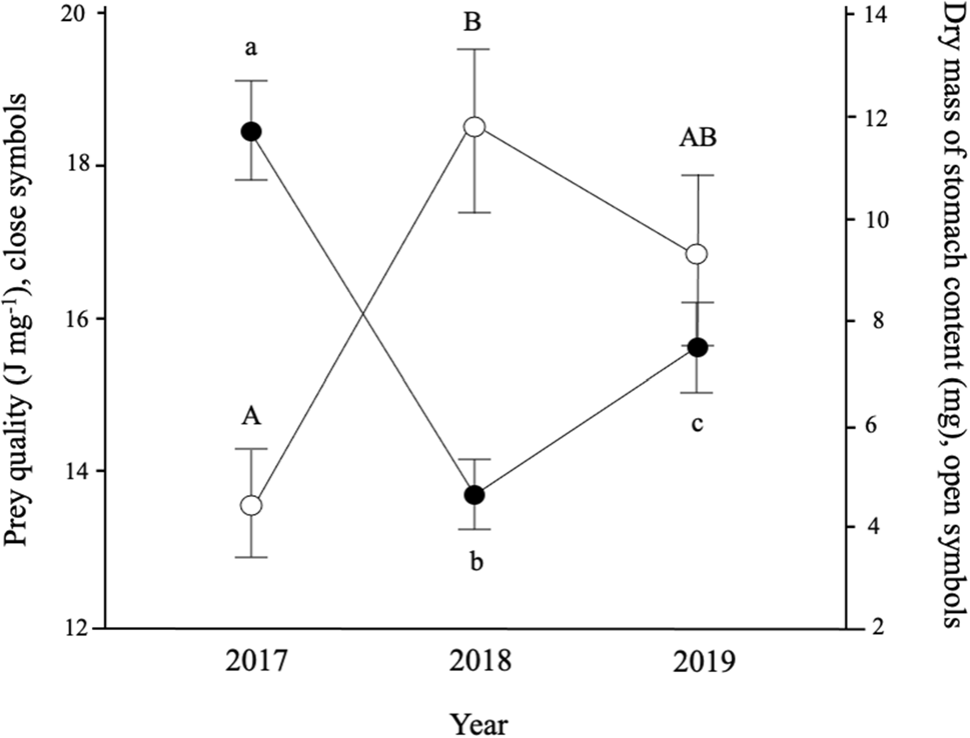
Variation in mean prey quality (close symbols) and dry mass of prey matter (open symbols) found in nestling’s stomachs over 3 years in black-capped chickadees and boreal chickadees sampled at the FER Macpès, Rimouski, Canada (data combined for both species, see text). We used data from 63 broods for these analyses. Of those, 39 broods were black-capped chickadees and 24 broods were boreal chickadees. Stomachs contained more dry matter in years of low food quality. Different letters (lower case for prey quality and upper case for stomach contents) indicate significant differences among years. Data are mean ± 95% confidence interval.

Prey quality also varied significantly among years (F_2,54_ = 13.43, P < 0.001), being 31% higher in 2017 (17.97 ± 3.84 J mg^−1^, Fig. 3) than in 2018 (13.68 ± 3.49 J mg^−1^, Fig. 3) (Tukey: F_1,56_ = 26.84, P < 0.001). It did not vary over time within years, however, (Julian date: F_1, 58_ = 1.68, P = 0.2), nor with nestling age (F_2,129_ = 2.76, P = 0.07) or brood size (F_7, 63_ = 1.83, P = 0.09). The amount of prey found in nestling stomachs (dry mass) also varied significantly among years (F_2, 55_ = 5.33, P < 0.01) and showed an opposite pattern to prey quality (Fig. 3). In 2017, when prey quality was at its highest, stomachs contained the lowest amount of food (5.90 ± 7.70 mg). Inversely, in 2018, when food was at its lowest quality, dry mass of prey was at its highest, with parents bringing 2.1 times more food to their nestlings than in 2017 (12.40 ± 12.32 mg, Tukey: F_1,57_ = 10.63, P < 0.005, Fig. 3). Dry mass of stomach contents did not vary over time within years (Julian date: F_1,59_ = 6.00^e−04^, P = 0.9) but changed with the age of nestlings (F_2, 131_ = 12.44, P < 0.001), being 110% higher on days 10 and 12 (mean 12.56 ± 11.33 mg per brood, no significant difference, Tukey : F_1,125_ = 0.64, P = 0.7) compared to day 8 (5.99 ± 5.98 mg, Tukey: F_1,146_ = 20.84, P < 0.001). Not surprisingly, as our samples pooled stomach contents from all nestlings in a brood, brood size also affected dry mass of stomach contents (F _7,66_ = 2.30, P < 0.05). Stomachs in broods of 3 contained an average of 5.67 ± 10.93 mg dry mass of food compared to 10.44 ± 9.18 mg in a brood of 5. However, this effect remained statistically weak, since a post-hoc Tukey test could not detect any significant differences among means (Tukey: F_1,57_ = 2.49, P = 0.7).

### Inter-relation between growth rate, provisioning rate and prey quality across years

**Figure 4:**
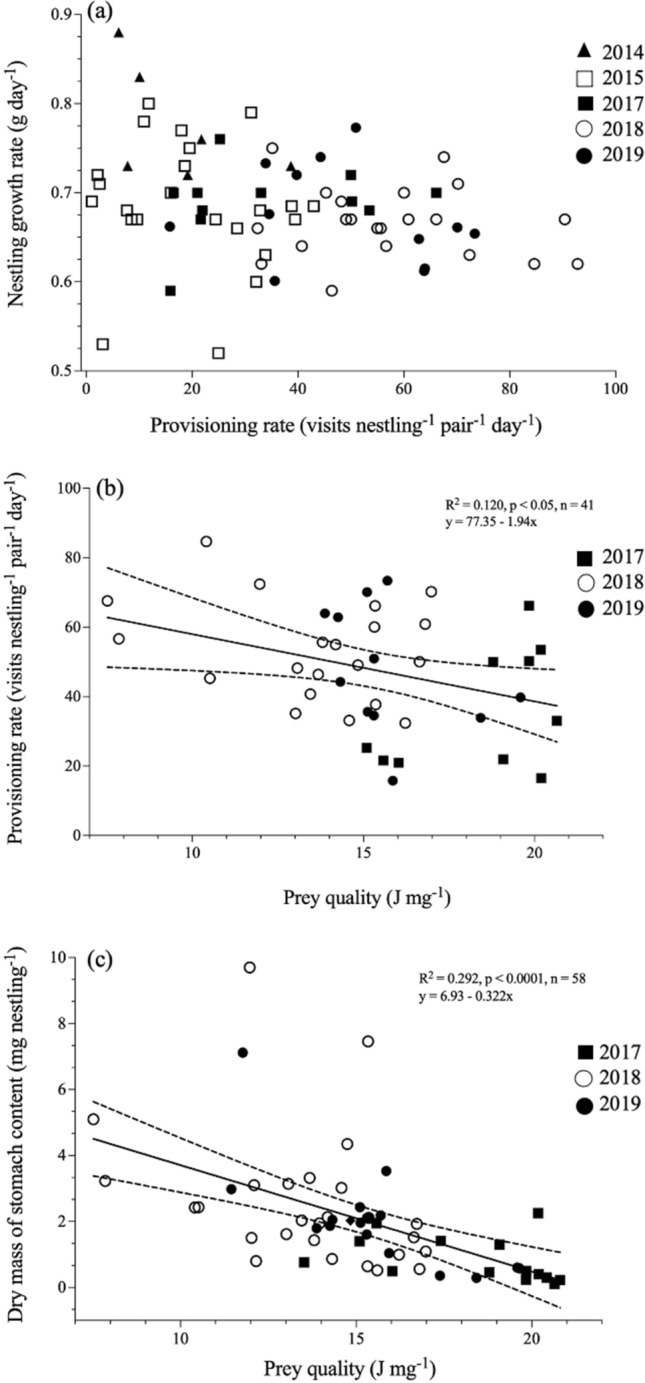
Linear regressions between provisioning rate and growth rate (**a**, 73 broods, including 39 of black-capped chickadees and 34 of boreal chickadees), between prey quality and provisioning rate (**b**, 41 broods, 24 of black-capped chickadees and 17 of boreal chickadees) and between prey quality and dry mass of stomach contents (**c**, 58 broods, 36 of black-capped chickadees and 22 of boreal chickadees) in nestlings of black-capped and boreal chickadees sampled at the FER Macpès, Rimouski, Canada (data combined for both species, see text). Growth rate varied independently from provisioning rate, but birds provisioned more often when food was of lower quality. More food was also found in nestling stomachs when food was of lower quality. See text for full mixed model results including all the variables. The dotted lines show 95% confidence intervals for significant regression models.

Nestling growth rate was not significantly related to provisioning rate (F_1, 63_ = 2.48, P = 0.1, Fig. 4a, brood size: F_7,61_ = 3.02, P < 0.01), or to prey quality (F_1, 24_ = 0.03, P = 0.9, brood size: F_6, 49_ = 8.37, P < 0.001) across years. However, provisioning rate varied negatively with the quality of food found in nestling stomachs (F_1, 34_ = 15.80, P < 0.001, Fig. 4b, brood size: F_5, 34_ = 0.69, P < 0.001). Similarly, nestlings had more food in their stomachs when prey quality was low (F_1, 50_ = 27.08, P < 0.001, Fig. 4c, brood size: F_6, 50_ = 1.36 , P = 0.2).

## Discussion

In this study, we monitored breeding and the provisioning behavior of black-capped and boreal chickadees over a period spanning 8 years. We found no support for an influence of provisioning rate, measured as the number of daily visits by breeding pairs, on nestling growth rate. We also found that the mean daily growth rate was consistent across years, suggesting that growth is maintained despite marked variation in prey quality among years. However, the provisioning rate did differ across years, being twice as high in 2018, a low prey quality year, as in 2017 when prey quality was high, suggesting that adults adjust foraging and provisioning efforts to prey quality. Indeed, variation in provisioning rate and the amount of food provided to nestlings correlated negatively with prey quality across years, and this most likely explained the lack of a relationship between offspring growth rate and parental provisioning rate.

Comparing years, we found that the average nestling growth rate was consistent over time, despite considerable yearly variation in prey quality and provisioning rates. This constant growth rate supports the idea that growth is maximized to achieve early nest departure in nestlings^38–40^. Nevertheless, our data also showed a large variation in prey energy content across years, likely reflecting yearly variation in abiotic factors (e.g., temperature), which can influence arthropod development^7,37^. Previous studies have shown that breeding birds can respond spatially (e.g., foraging adjusted to arthropod distribution) and temporarily (e.g., the timing of breeding. cf.^41–44^) to variation in the abundance and size of prey^1,2,7,9,39,45^. Some bird species also appear to maintain nestling growth by reducing brood size in poor years^10,46^. In chickadees, brood size at fledging did not vary among years, but we found that the provisioning rate was 60% higher in 2018 than in 2017. In 2018, the prey found in nestlings’ stomachs contained only 76% of the energy measured in 2017 and nestling stomachs contained twice as much prey matter compared to 2017. This is consistent with previous studies showing higher provisioning rates in birds exploiting low quality food sources^47–49^. To our knowledge, this constitutes the first demonstration of this phenomenon at an interannual scale and in Paridae of the New World. Clearly, both chickadee species were able to compensate for lower prey quality to maintain the growth rate of nestlings.

When investigating the inter-relation between growth rate, provisioning rate and prey quality across years, we found that growth rate was not related to variation in provisioning rate. This lack of relationship has been reported several times before^8,12–15,20,50^ and is consistent with breeding adults adjusting their provisioning effort to prey quality. Indeed, chickadees were provisioning nestlings at higher rates when prey contained less energy per unit mass and nestling stomachs contained more dry matter when food had lower energy content. Our study, therefore, supports our hypothesis that nestling growth rates are independent of provisioning rates due to parental behavioral adjustment in response to prey quality. This reasoning is also compatible with previous observations in blue tits (*Cyanistes caeruleus*) where provisioning rates did not differ between breeding sites of different quality^50^. However, birds provisioning nestlings in a low-quality habitat had to compensate by flying greater distances to forage and maintain nestling growth compared to those breeding in a high-quality habitat^50^.

We predicted that nestling growth rate should also correlate with prey quality but found a non-significant relationship between these variables. However, although it is ultimately the daily energy and nutrient intake by nestlings that should be the primary driver of growth^40,51^, our technique was likely not optimal for measuring that specific relationship. Stomach energy contents were obtained over three single events per brood, representing the energy available to nestlings at those specific time points within a sampling day. Although the approach is valid for measuring prey quality based on energy content, it does not provide detailed information on total energy delivered to nestlings for those specific sampling days or the entire growth period. Therefore, it appears that the amount of energy delivered predominates over prey quality in determining growth rate, which forces adults to increase provisioning rates in years of low prey quality to maintain nestling growth rates.

Two mechanisms could allow adult chickadees to adjust provisioning rates to prey quality. Parents consuming the same prey as their nestlings (Senécal et al. pers. obs.) could detect prey quality themselves and adjust their food intake and food delivery to nestlings in response to prey quality. For example, experimental studies have shown increases in food consumption when food items have lower digestibility^52–54^. Alternatively, but not exclusively, parents could adjust food delivery to the begging levels of nestlings. Begging intensity is related to hunger^55,56^ and is considered an honest signal of nestling requirements^57^. Thus, parents usually feed their nestlings proportionally to the intensity of begging^58–60^. Consequently, nestlings in poorer conditions that beg at higher rates should receive more food than nestlings in better condition^60–66^. Hence, begging should be higher in years of low food quality in chickadees.

This study shows that variation in prey quality drives breeding chickadees to adjust their provisioning rate to maintain nestling growth. This leads to a lack of a relationship between growth rate and provisioning rate. Therefore, although the provisioning rate should still reflect breeding effort through locomotion costs (see^67^), it cannot be interpreted as an indicator of the amount of energy delivered through food to nestlings.

## Supplementary Information

Supplementary Information.

## Data Availability

The datasets used for this study are available from the corresponding author upon request.
