# Supplementary material for: Poor prey quality is compensated by higher provisioning effort in passerine birds
Source: Sci Rep. 2021 May 27;11:11182. doi: 10.1038/s41598-021-90658-w (PMC8159977; doi:10.1038/s41598-021-90658-w)
Supplement: Supplementary file 1 — Supplementary Information. [file 41598_2021_90658_MOESM1_ESM.docx]

**Poor prey quality is compensated by higher provisioning effort in passerine birds**

**—Online Supplementary Material—**

Sarah Senécal, Julie-Camille Riva, Ryan S. O’Connor, Fanny Hallot, Christian Nozais & François Vézina

**Content**

Provisioning data…………………………………………………………………….Page 2

Patterns of growth in black-capped and boreal chickadees………………..………..Page 4

Variation in provisioning rate, prey quality and dry mass between species.………..Page 7

**Provisioning data**

In our study, most breeding adults were ringed on day 13 (day 1 being the hatching day), since captures of adults before this day increase the risk of nest abandonment. If breeding pairs included at least one adult already ringed with a PIT-tag from a previous year, the RFID system was installed on the nest box on day 1 or as soon as the adult was identified.

To confirm that our measure of provisioning rate after day 13 reflected that of the whole nestling growth period, we calculated repeatability of individual daily provisioning rate by comparing measurements taken before and after day 13 in pairs for which this data was available for at least one bird. We modelled variation in provisioning rate (per individual adult) by fitting a linear mixed-effect model with brood size and nestling age as fixed effects and adult identification number as well as pair identity as random factors. We used data from 101 individuals from 60 pairs (17 pairs with data for both adults from day 1). Provisioning rate increased with nestling age (F_1, 534_ = 5.93, P <0.05), and with brood size (F_1, 144_ = 33.14, P <0.001). As shown in Table S1, the individual (Breeder ID) explained 52% of the total variance.

**Table S1**. Variation in provisioning rate per individual within year

| Random factor | Variance | % of variance explained | Standard deviation |
| --- | --- | --- | --- |
| Breeder ID | **10940** | 52 | 104.60 |
| Pair ID | 3091 | 14 | 55.60 |
| Residual | 7092 | 34 | 84.21 |


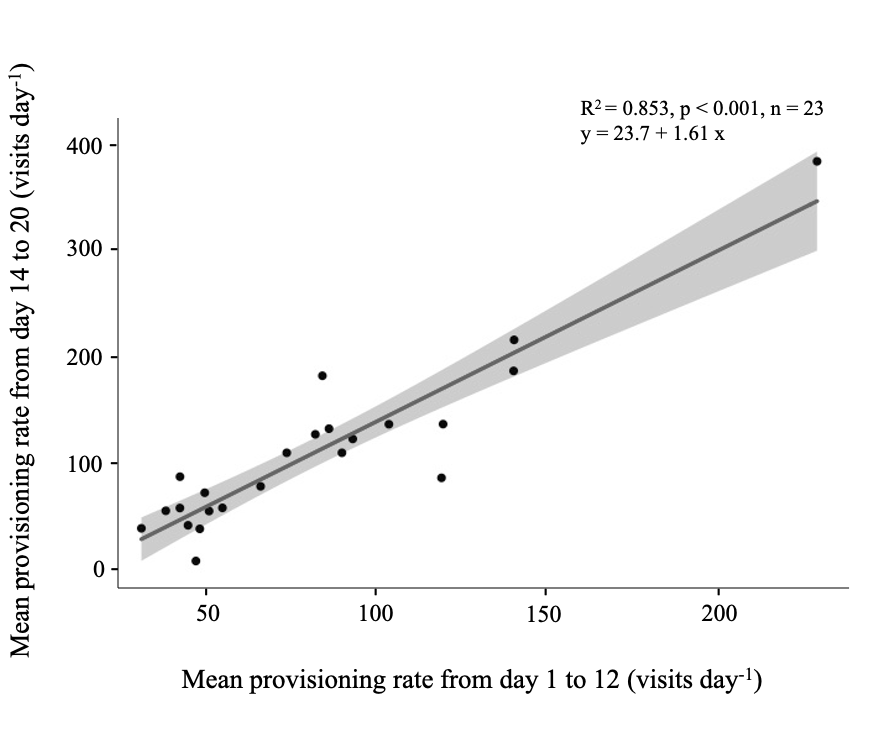
To validate the use of a mean number of visits per day per breeding pairs, we also tested for a correlation between mean daily provisioning rate measured when nestlings were 1 to 12 days old and mean daily provisioning rate from day 14 to day 20 (adults were captured on day 13). This simple linear regression analysis included individuals from 17 pairs (Figure S1).

**Figure S1**. Provisioning rate of breeding black-capped and boreal chickadees comparing rates between days 1 to 12 to those recorded between days 14 to 20 (dots represents breeding individuals). The grey bands show 95% confidence intervals.

Provisioning rate increased with nestlings age but is highly repeatable among periods (R^2^ = 0.853, F_1, 21_ = 121.9, P < 0.001). Note that removing the extreme high value point from Figure S1 does not change the outcome of this analysis.

In this study, we pooled the species data to avoid duplication of results because nestling mass and growth rate patterns as well as parental provisioning rate and stomach content (quality and dry mass) did not differ significantly between the two species.

**Patterns of growth in black-capped and boreal chickadees**

Preliminary analyses revealed very close patterns of nestling mass and growth rates from day 1 to 15 in black-capped and boreal chickadees. These analyses used linear mixed-effect models to test whether nestling mass (Table S2) or growth rate (Table S3) differed according to species, while considering the effect brood size (1-8 nestlings) as a fixed factor, where year (2011-2019, excluding 2016 where a separate investigation including brood manipulation took place) and pair identity were included as random factors. This included data for 858 nestlings from 173 broods (98 black-capped chickadees, 75 boreal chickadees). Results showed very similar patterns of growth in both species. Daily masses and growth rates per species are presented here for reference (Figure S2 and S3).

**Table S2.** ANOVA table of the model for nestling mass

| Fixed effect | | | |
| --- | --- | --- | --- |
|  | F-value | DF | P-value |
| Species | 3.6058 | 1, 2188 | 0.09771 |
| Brood size | 2.2368 | 7, 2162 | 0.02883 |

**Table S3.** ANOVA table of the model for nestling growth rate

| Fixed effect | | | |
| --- | --- | --- | --- |
|  | F-value | DF | P-value |
| Species | 2.4697 | 1, 2184 | 0.1162 |
| Brood size | 7.4300 | 7, 2184 | 7.461e-09 |

**
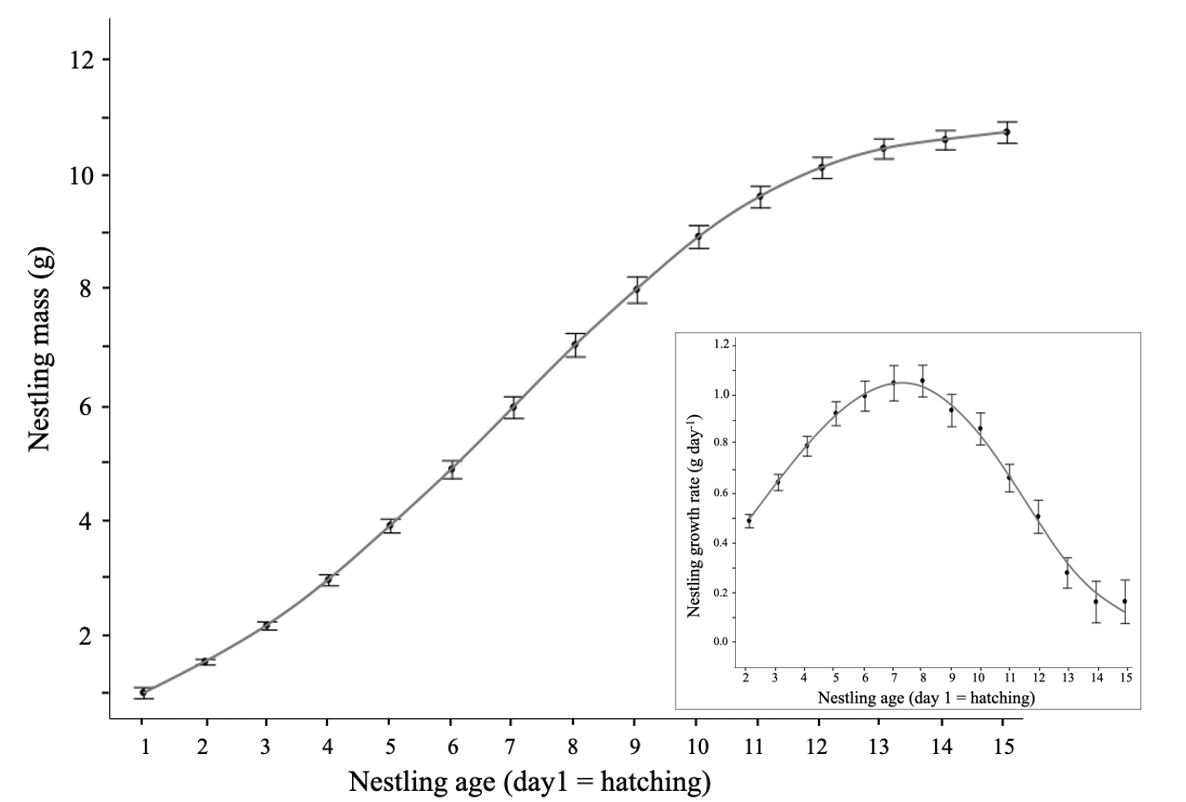
**

**Figure S2**: Growth curve showing nestling mean daily mass according to age in days since hatching in black-capped chickadees (*Poecile atricapillus*) sampled at the FER Macpès, Rimouski, Canada. Insert shows daily growth rate (calculated over the last 24h) according to nestling age. We used data from 98 broods for this analysis. Data are mean ± 95% confidence interval.

**
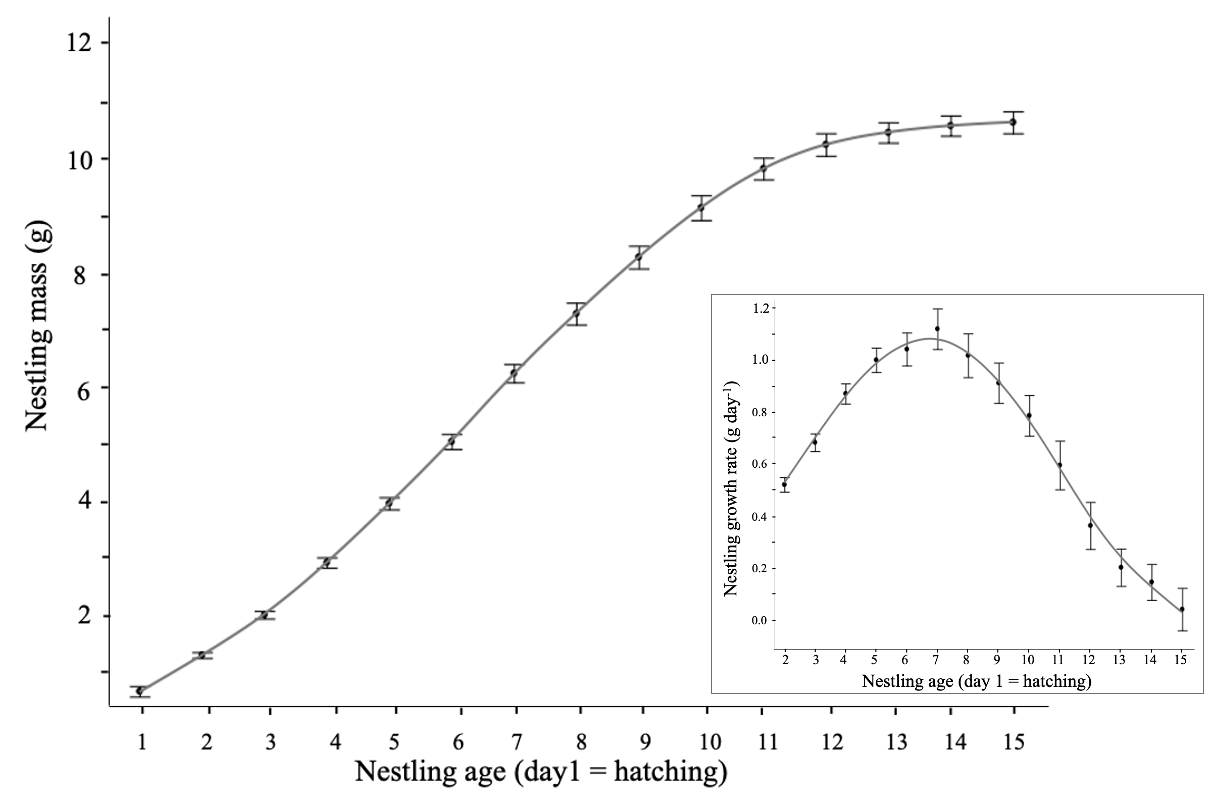
**

**Figure S3**: Growth curve showing nestling mean daily mass according to age in days since hatching in boreal chickadees (*Poecile hudsonicus*) sampled at the FER Macpès, Rimouski, Canada. Insert shows daily growth rate (calculated over the last 24h) according to nestling age. We used data from 75 broods for this analysis. Data are mean ± 95% confidence interval.

**Variation in provisioning rate, prey quality and dry mass between species**

We show here ANOVA tables from models we ran including species as a fixed effect to confirm that the patterns did not differ between species. These analyses used multiple regression models and tested if provisioning rate (Table S4), prey quality (Table S5) or dry mass of stomach content (Table S6) differed between species. Models included the effects of species and brood size as fixed factors. For the provisioning rate analysis (Table S4), this included data from 73 broods (39 black-capped chickadees, 34 boreal chickadees). For prey quality (Table S5) and dry mass (Table S6), analysis, this included data from 58 broods (39 black-capped chickadees, 34 boreal chickadees). Results showed undistinguishable patterns in both species.

**Table S4.** ANOVA table of the model for provisioning rate

| Fixed effect | | | |
| --- | --- | --- | --- |
|  | F-value | DF | P-value |
| Species | 0.7535 | 1 | 0.3915 |
| Brood size | 0.7780 | 5 | 0.5724 |

**Table S5.** ANOVA table of the model for prey quality

| Fixed effect | | | |
| --- | --- | --- | --- |
|  | F-value | DF | P-value |
| Species | 0.0006 | 1 | 0.97980 |
| Brood size | 1.9458 | 6 | 0.09153 |

**Table S6.** ANOVA table of the model for dry mass of stomach content

| Fixed effect | | | |
| --- | --- | --- | --- |
|  | F-value | DF | P-value |
| Species | 1.9377 | 1 | 0.1701 |
| Brood size | 1.4245 | 6 | 0.2240 |
